# Prospective serum metabolomic profile of prostate cancer by size and extent of primary tumor

**DOI:** 10.18632/oncotarget.16775

**Published:** 2017-04-01

**Authors:** Jiaqi Huang, Alison M. Mondul, Stephanie J. Weinstein, Edward D. Karoly, Joshua N. Sampson, Demetrius Albanes

**Affiliations:** ^1^ Division of Cancer Epidemiology and Genetics, National Cancer Institute, NIH, Department of Health and Human Services, Bethesda, MD, USA; ^2^ Department of Epidemiology, University of Michigan School of Public Health, Ann Arbor, MI, USA; ^3^ Metabolon, Inc., Morrisville, NC, USA

**Keywords:** metabolomics, prostate cancer, biomarkers, tumor size T2/T3/T4

## Abstract

Two recent investigations found serum lipid and energy metabolites related to aggressive prostate cancer up to 20 years prior to diagnosis. To elucidate whether those metabolomic profiles represent etiologic or tumor biomarker signals, we prospectively examined serum metabolites of prostate cancer cases by size and extent of primary tumors in a nested case-control analysis in the ATBC Study cohort that compared cases diagnosed with T2 (*n* = 71), T3 (*n* = 51), or T4 (*n* = 15) disease to controls (*n* = 200). Time from fasting serum collection to diagnosis averaged 10 years (range 1–20). LC/MS-GC/MS identified 625 known compounds, and logistic regression estimated odds ratios (ORs) associated with one-standard deviation differences in log-metabolites. N-acetyl-3-methylhistidine, 3-methylhistidine and 2′-deoxyuridine were elevated in men with T2 cancers compared to controls (ORs = 1.38–1.79; 0.0002 ≤ *p* ≤ 0.01). By contrast, four lipid metabolites were inversely associated with T3 tumors: oleoyl-linoleoyl-glycerophosphoinositol (GPI), palmitoyl-linoleoyl-GPI, cholate, and inositol 1-phosphate (ORs = 0.49–0.60; 0.000017 ≤ *p* ≤ 0.003). Secondary bile acid lipids, sex steroids and caffeine-related xanthine metabolites were elevated, while two Krebs cycle metabolites were decreased, in men diagnosed with T4 cancers. Men with T2, T3, and T4 prostate cancer primaries exhibit qualitatively different metabolite profiles years in advance of diagnosis that may represent etiologic factors, molecular patterns reflective of distinct primary tumors, or a combination of both.

## INTRODUCTION

As the second most common malignancy in men worldwide [1], the primary prevention of prostate cancer is important yet hampered by the lack of well-established modifiable risk factors for the disease. Untargeted metabolomic approaches have been increasingly employed to identify a broad array of low molecular weight compounds potentially related to cancer etiology, early detection and progression, and that may help elucidate underlying biologic mechanisms [2, 3].

Two studies in the Alpha-Tocopherol, Beta-Carotene Cancer Prevention (ATBC) Study cohort prospectively examined serum metabolomic profiles of prostate cancer up to two decades prior to diagnosis [4, 5]. Case-control differences in circulating lipid and energy metabolites, including alpha-ketoglutarate, citrate, inositol-1-phosphate, glycerophospholipids, and fatty acids were identified [4, 5]. Just as distinct metabolite signals were observed for aggressive versus non-aggressive prostate malignancies in these studies, metabolomic patterns may exist that reflect underlying tumor biology related to other clinical characteristics. The present analysis was undertaken in order to test whether the serum metabolite profiles of men diagnosed with T2, T3 and T4 prostate cancers within 20 years of blood sampling qualitatively differ from each other.

## RESULTS

Baseline characteristics of the cases and controls are presented in Table 1. Most characteristics were similar across the groups with the exception of serum total PSA, with substantially higher concentrations in each primary tumor category of cases, compared with controls. All of the characteristics were similar among T2, T3 and T4 cases, with only one exception being physical activity, which might be due to chance based on the small number of T4 cases. Median age at diagnosis was 68 years.

**Table 1:** Baseline characteristics of cases and controls^1^

	Controls	T2 cases		T3 cases		T4 cases		*P*^2^
			*P*		*P*		*P*	
*N*	200	71	--	51	--	15	--	
Age (years)	59.3	59.8	0.6	58	0.09	60.9	0.3	0.09
Median time from serum collection to diagnosis (years, interdecile range)	--	8.1 (1.9–16.9)	--	10.9 (3.3–18.3)	--	8.2 (2.1–16.2)	--	0.18
Height (cm)	173.1	172.8	0.89	174.1	0.23	173.9	0.54	0.52
Weight (kg)	78.1	80.9	0.17	78.3	0.93	82.8	0.21	0.37
BMI (kg/m^2^)	26	27	0.1	25.8	0.52	27.4	0.22	0.15
Family history of prostate cancer (%)	3.5	5.3	0.61	15.2	0.009	0	--	0.22
Cigarettes per day	19.4	18.4	0.31	19.8	0.82	18.2	0.56	0.63
Years of cigarette smoking	37.5	38.3	0.42	36.2	0.44	41.7	0.08	0.11
Physically active (%)	19	5.6	0.008	21.6	0.68	0	--	0.007
Serum total PSA (ng/mL)	1.4	8.9	0.003	8.1	0.001	10.7	0.038	0.79
Serum retinol (μg/L)	587	597	0.96	611	0.53	588	0.79	0.73
Serum total cholesterol (mmol/L)	6.2	6.2	0.91	6.1	0.72	6	0.51	0.83
Serum α-tocopherol (mg/L)	11.8	12	0.49	11.2	0.24	11.9	0.85	0.27
Serum β-carotene (μg/L)	200	197	0.99	220	0.22	217	0.67	0.64
Dietary intake per day								
Total energy (kcal)	2667	2689	0.95	2553	0.22	2391	0.14	0.36
Fruit (g)	216	202	0.42	201	0.53	127	0.004	0.24
Vegetables (g)	293	297	0.92	264	0.07	271	0.43	0.23
Red meat (g)	70.5	72	0.98	62.1	0.052	69.3	0.76	0.18
Alcohol (ethanol, g)	17.4	18.7	0.63	13.8	0.17	17.7	0.56	0.37
Supplement use								
Vitamin A (%)	10.6	11.4	0.85	9.8	0.87	13.3	0.74	0.92
Vitamin D (%)	7.1	5.7	0.7	5.9	0.76	6.7	0.95	0.99
Calcium (%)	13.6	12.9	0.87	11.8	0.73	6.7	0.44	0.8

^1^Values are means unless otherwise indicated. All characteristics were obtained at baseline, except family history of prostate cancer, which was collected during follow-up and is available for 223 men in the analysis.

^2^*P* value for difference among T2, T3 and T4 cases, using ANOVA test (the analysis of variance) for continuous, and chi-squared tests for categorical variables, respectively.

Using a *p* value threshold of < 0.05 for either T2, T3 or T4 prostate cancers, we show that metabolites associated with T2, T3 and T4 prostate cancers (Tables 2 and 3, sorted by chemical class and subclass, with lipids shown in Table 3). As compared to the control group, men with T2 prostate cancers had elevated serum amino acids in histidine metabolism including N-acetyl-3-methylhistidine (OR= 1.79, *p* = 0.0002; FDR of < 20%), 3-methylhistidine (p=0.01), imidazole lactate (*p* = 0.03), and nucleotide 2′-deoxyuridine (*p* = 0.005), and they had lower glycerophospholipids stearoyl-arachidonoyl-glycerophosphoethanolamine (GPE) (*p* = 0.02), and stearoyl-linoleoyl-GPE (*p* = 0.03) lipid compounds, and two benzoate metabolites (3-ethylphenylsulfate and 2-ethylphenylsulfate) (Tables 2 and 3).

**Table 2:** Serum metabolites associated with T2, T3 and T4 prostate cancers ^1,2,3^

	T2					T3				T4				*P* het^4^
Variable	CS^5^	OR	95% CI		*P*	OR	95% CI		*P*	OR	95% CI		*P*	
**Amino acids**														
N-Acetylmethionine	A-1	0.94	0.72	1.23	0.632	1.22	0.86	1.71	0.264	**0.65**	**0.43**	**0.99**	**0.047**	0.041
N-Acetylglutamine	A-2	1.06	0.82	1.38	0.642	0.97	0.70	1.34	0.867	**0.52**	**0.28**	**0.96**	**0.038**	0.062
Cysteine-glutathione disulfide	A-2	0.98	0.75	1.29	0.904	1.29	0.92	1.81	0.141	**0.59**	**0.39**	**0.89**	**0.012**	0.008
4-Imidazoleacetate	A-3	1.28	0.95	1.74	0.107	**1.59**	**1.11**	**2.3**	**0.013**	**2.52**	**1.25**	**5.12**	**0.010**	0.335
Imidazole lactate	A-3	**1.33**	**1.02**	**1.74**	**0.033**	0.99	0.73	1.35	0.957	0.98	0.58	1.65	0.939	0.146
3-Methylhistidine	A-3	**1.38**	**1.07**	**1.79**	**0.012**	0.80	0.57	1.13	0.208	1.00	0.61	1.64	0.999	0.0058
N-Acetyl-3-methylhistidine^3^	A-3	**1.79**	**1.31**	**2.44**	**0.00024**	0.78	0.57	1.08	0.134	0.95	0.57	1.59	0.855	0.00009
O-Cresol sulfate	A-4	**0.76**	**0.59**	**0.99**	**0.039**	1.09	0.79	1.5	0.601	0.90	0.54	1.50	0.676	0.184
Indoleacetate	A-5	1.05	0.78	1.41	0.756	1.10	0.79	1.54	0.564	**1.89**	**1.11**	**3.20**	**0.019**	0.300
5-Hydroxyindoleacetate	A-5	1.26	0.91	1.73	0.159	**1.49**	**1.01**	**2.19**	**0.042**	0.95	0.51	1.78	0.879	0.457
N-Acetylarginine	A-6	1.25	0.96	1.63	0.092	0.96	0.71	1.31	0.814	**0.54**	**0.31**	**0.93**	**0.028**	0.015
N-Acetylcitrulline	A-6	**1.37**	**1.03**	**1.82**	**0.028**	1.16	0.84	1.6	0.368	0.97	0.60	1.59	0.911	0.334
β-hydroxyisovalerate	A-7	**1.48**	**1.07**	**2.05**	**0.019**	1.07	0.80	1.44	0.632	1.68	0.85	3.29	0.133	0.382
2-Hydroxyisobutyrate	A-7	0.93	0.71	1.22	0.598	0.86	0.62	1.20	0.378	**0.44**	**0.23**	**0.84**	**0.013**	0.136
**Cofactors and vitamins**														
L-Urobilin	C-1	**1.35**	**1.03**	**1.76**	**0.031**	0.99	0.71	1.37	0.930	0.96	0.55	1.65	0.874	0.129
**Energy**														
Citrate	E-1	0.98	0.75	1.30	0.906	0.89	0.65	1.21	0.444	**0.56**	**0.32**	**0.99**	**0.045**	0.086
Fumarate	E-1	1.01	0.78	1.31	0.942	1.07	0.79	1.44	0.681	**0.55**	**0.33**	**0.93**	**0.024**	0.0069
**Nucleotides**														
Orotate	N-1	0.98	0.75	1.29	0.895	1.23	0.89	1.69	0.207	**0.40**	**0.21**	**0.74**	**0.0039**	0.0046
5,6-Dihydrothymine	N-1	0.99	0.74	1.34	0.964	0.90	0.67	1.21	0.496	**0.58**	**0.34**	**0.99**	**0.047**	0.313
2′-Deoxyuridine	N-1	**1.71**	**1.18**	**2.47**	**0.0046**	1.38	0.93	2.04	0.115	1.49	0.76	2.93	0.250	0.630
**Peptides**														
γ-glutamyltryptophan	P-1	**1.43**	**1.04**	**1.97**	**0.027**	1.18	0.83	1.68	0.353	0.95	0.57	1.59	0.845	0.280
**Xenobiotics**														
3-Ethylphenylsulfate	X-1	**0.70**	**0.55**	**0.90**	**0.0047**	1.09	0.78	1.51	0.613	1.15	0.62	2.12	0.665	0.023
2-Ethylphenylsulfate	X-1	**0.77**	**0.60**	**0.98**	**0.034**	1.16	0.81	1.67	0.413	0.85	0.52	1.38	0.510	0.071
2-Aminophenol sulfate	X-2	0.93	0.71	1.21	0.577	**0.71**	**0.52**	**0.96**	**0.025**	1.08	0.64	1.82	0.771	0.154
Salicyluric glucuronide	X-3	0.90	0.69	1.19	0.465	**0.66**	**0.45**	**0.96**	**0.031**	0.92	0.56	1.52	0.749	0.502
2-Acetamidophenol sulfate	X-3	0.94	0.72	1.24	0.670	**0.72**	**0.54**	**0.97**	**0.032**	1.00	0.60	1.67	0.999	0.115
S-Allylcysteine	X-4	0.87	0.64	1.18	0.378	1.00	0.74	1.36	0.990	**1.60**	**1.06**	**2.41**	**0.025**	**0.046**
Isoeugenol sulfate	X-4	0.93	0.71	1.21	0.585	**1.55**	**1.06**	**2.25**	**0.023**	1.08	0.61	1.91	0.797	0.117
Paraxanthine	X-5	0.96	0.70	1.30	0.772	0.86	0.64	1.16	0.318	**2.53**	**1.15**	**5.53**	**0.020**	0.089
Caffeine	X-5	0.98	0.74	1.31	0.912	0.87	0.63	1.2	0.399	**2.40**	**1.24**	**4.66**	**0.010**	0.037
Theobromine	X-5	0.98	0.72	1.34	0.916	0.86	0.65	1.15	0.312	**2.46**	**1.08**	**5.59**	**0.031**	0.151
Theophylline	X-5	0.98	0.72	1.32	0.881	0.93	0.67	1.28	0.647	**1.71**	**1.02**	**2.85**	**0.040**	0.138
1,3,7-Trimethylurate	X-5	1.03	0.78	1.36	0.830	0.93	0.68	1.28	0.665	**2.31**	**1.17**	**4.56**	**0.016**	0.084

Abbreviations: OR = odds ratio; CI = confidence interval.

^1^The odds ratio per standard deviation increase in metabolite, and estimated by unconditional logistic regression model adjusted for age. Based on T2 (*N* = 71), T3 (*N* = 51), T4 (*N* = 15), and 200 controls. The table is sorted by chemical class and subclass. We highlighted metabolites (ORs and 95% CIs) that achieved the nominal significance of *p* < 0.05 within each class.

^2^All metabolites had a detectable value in > 90% of the study population with the exception of 3-ethylphenylsulfate, 1,3,7-trimethylurate, N-acetyl-3-methylhistidine, L-urobilin, salicyluric glucuronide, S-allylcysteine (12%, 13%, 19%, 49%, 57%, 75%, respectively).

^3^N-Acetyl-3-methylhistidine in T2 cases achieved statistical significant with a false discovery rate (FDR) of < 20%.

^4^*P* heterogeneity: We tested whether the metabolite levels differed across extent of disease (i.e. heterogeneity) by case-only analyses. Modeling extent of disease (T2/T3/T4) by polychotomous logistic regression, the *p*-value (*p* heterogeneity) was calculated from a likelihood ratio test comparing models with and without the metabolite level.

^5^CS = Chemical subclass: (A- amino acids) A-1 = Cysteine, methionine, SAM, taurine metabolism; A-2 = Glutamate metabolism; A-3 = Histidine metabolism; A-4 = Phenylalanine and tyrosine metabolism; A-5 = Tryptophan metabolism; A-6 = Urea cycle; arginine and proline metabolism; A-7 = Valine, leucine and isoleucine metabolism; (C- cofactors and vitamins) C-1 = Hemoglobin and porphyrin metabolism; (E- energy) E-1 = Krebs cycle/TCA cycle; (N- nucleotides) N-1 = Pyrimidine metabolism, orotate containing; ( P- peptides) P-1 = Gamma-glutamyl amino acid; (X-xenobiotics) X-1 = Benzoate metabolism; X-2 = Chemical; X-3 = Drug; X-4 = Food component/plant; X-5 = Xanthine metabolism.

**Table 3:** Serum lipid metabolites associated with T2, T3 and T4 prostate cancers ^1,2,3^

	T2					T3				T4				*P* het^4^
Variable	CS^5^	OR	95% CI		*P*	OR	95% CI		*P*	OR	95% CI		*P*	
Hexanoylglycine	L-1	1.01	0.74	1.38	0.948	0.95	0.68	1.32	0.758	**0.58**	**0.38**	**0.91**	**0.018**	0.153
N-Linoleoylglycine	L-1	1.13	0.88	1.44	0.336	**0.51**	**0.27**	**0.97**	**0.042**	**1.29**	**0.85**	**1.95**	**0.225**	0.0038
17-Methylstearate	L-2	0.91	0.68	1.20	0.493	0.82	0.59	1.13	0.229	**0.50**	**0.29**	**0.88**	**0.016**	0.197
Maleate	L-3	0.92	0.68	1.24	0.576	1.00	0.74	1.36	0.988	**1.66**	**1.05**	**2.62**	**0.030**	0.178
9,10-DiHOME	L-4	0.80	0.62	1.05	0.108	1.13	0.81	1.59	0.469	**0.59**	**0.38**	**0.92**	**0.019**	0.0162
Inositol-1-phosphate	L-5	0.93	0.71	1.22	0.587	**0.60**	**0.43**	**0.84**	**0.003**	0.68	0.40	1.15	0.148	0.078
Stearoyl-arachidonoyl-GPE	L-6	**0.73**	**0.55**	**0.95**	**0.019**	**0.68**	**0.49**	**0.94**	**0.020**	1.07	0.61	1.90	0.807	0.319
Stearoyl-linoleoyl-GPE	L-6	**0.73**	**0.55**	**0.96**	**0.025**	**0.64**	**0.45**	**0.93**	**0.018**	1.29	0.70	2.36	0.412	0.202
Oleoyl-linoleoyl-GPI^3^	L-6	0.88	0.67	1.15	0.349	**0.49**	**0.35**	**0.68**	**0.000017**	1.19	0.66	2.17	0.562	0.0041
1-Stearoyl-GPG	L-6	0.94	0.72	1.23	0.649	**0.69**	**0.50**	**0.96**	**0.029**	1.48	0.89	2.47	0.135	0.011
Palmitoyl-linoleoyl-GPI	L-6	0.98	0.75	1.29	0.908	**0.56**	**0.39**	**0.80**	**0.0012**	1.30	0.74	2.30	0.363	0.031
1-Linoleoyl-GPI	L-6	0.99	0.76	1.30	0.966	**0.70**	**0.51**	**0.95**	**0.024**	1.31	0.76	2.26	0.336	0.154
2-Linoleoylglycerol	L-7	0.92	0.69	1.22	0.547	**0.73**	**0.53**	**1.00**	**0.047**	1.30	0.73	2.33	0.373	0.221
Cholate	L-8	1.01	0.76	1.34	0.952	**0.57**	**0.40**	**0.81**	**0.0019**	0.83	0.48	1.43	0.499	0.012
Chenodeoxycholate	L-8	1.09	0.82	1.46	0.549	**0.71**	**0.52**	**0.97**	**0.031**	0.77	0.48	1.25	0.291	0.042
Tauroursodeoxycholate	L-9	0.98	0.74	1.29	0.881	0.65	0.42	1.00	0.051	**1.61**	**1.08**	**2.39**	**0.019**	0.0029
Taurodeoxycholate	L-9	1.08	0.81	1.44	0.598	0.98	0.71	1.34	0.900	**2.62**	**1.30**	**5.27**	**0.0068**	0.016
Deoxycholate	L-9	1.09	0.82	1.44	0.557	0.97	0.71	1.32	0.858	**3.31**	**1.24**	**8.81**	**0.016**	0.0026
Glycodeoxycholate	L-9	1.17	0.88	1.56	0.269	0.92	0.68	1.24	0.575	**3.19**	**1.38**	**7.38**	**0.0068**	0.0068
Glycolithocholate sulfate	L-9	**1.37**	**1.03**	**1.81**	**0.029**	1.35	0.98	1.86	0.066	**1.83**	**1.02**	**3.28**	**0.043**	0.361
Myristoyl sphingomyelin	L-10	1.03	0.79	1.34	0.825	**1.43**	**1.02**	**1.99**	**0.036**	0.76	0.46	1.26	0.289	0.114
Stearoyl sphingomyelin	L-10	1.13	0.86	1.50	0.381	**1.54**	**1.10**	**2.14**	**0.012**	1.00	0.58	1.71	0.988	0.166
Euricoyl sphingomyelin	L-10	**1.48**	**1.06**	**2.08**	**0.022**	**1.66**	**1.08**	**2.55**	**0.020**	1.37	0.71	2.64	0.347	0.817
Estrone 3-sulfate	L-11	1.15	0.88	1.50	0.293	0.78	0.55	1.10	0.150	**1.93**	**1.16**	**3.21**	**0.012**	0.016
4-Androsten-3alpha,17alpha-diol monosulfate	L-11	1.18	0.90	1.55	0.233	1.06	0.77	1.44	0.735	**2.00**	**1.04**	**3.84**	**0.038**	0.081
7-HOCA	L-11	1.21	0.92	1.60	0.177	1.03	0.75	1.44	0.840	**1.71**	**1.04**	**2.81**	**0.036**	0.267
5Alpha-androstan-3alpha,17alpha-diol disulfate	L-11	1.24	0.94	1.64	0.133	**0.68**	**0.49**	**0.93**	**0.017**	1.58	0.90	2.77	0.114	0.001
5Alpha-pregnan-3beta,20alpha-diol disulfate	L-11	1.27	0.96	1.68	0.099	1.07	0.78	1.47	0.691	**1.90**	**1.11**	**3.27**	**0.020**	0.098

Abbreviations: OR = odds ratio; CI = confidence interval; GPE = glycerophosphocholine; GPI = glycerophosphoinositol; GPG=glycerophosphoglycerol.

^1^The odds ratio per standard deviation increase in metabolite, and estimated by unconditional logistic regression model adjusted for age. Based on T2 (*N* = 71), T3 (*N* = 51), T4 (*N* = 15), and 200 controls. The table is sorted by chemical subclass. We highlighted metabolites (ORs and 95% CIs) that achieved the nominal significance of *p* < 0.05 within each subclass.

^2^All metabolites had a detectable value in > 90% of the study population with the exception of taurodeoxycholate, glycodeoxycholate, 5alpha-androstan-3alpha,17alpha-diol disulfate, 1-stearoylglycerophosphoglycerol, estrone 3-sulfate, tauroursodeoxycholate, N-linoleoylglycine (16%, 21%, 29%, 46%, 62%, 69%, 84%, respectively).

^3^Oleoyl-linoleoyl-GPI in T3 cases achieved the statistical significance after Bonferroni correction for multiple comparison.

^4^*P* heterogeneity: We tested whether the metabolite levels differed across extent of disease (i.e. heterogeneity) by case-only analyses. Modeling extent of disease (T2/T3/T4) by polychotomous logistic regression, the *p*-value (*p* heterogeneity) was calculated from a likelihood ratio test comparing models with and without the metabolite level.

^5^CS = Chemical subclass (L- lipid): L-1 = Fatty acid metabolism (acyl glycine); L-2 = Fatty acid, branched; L-3 = Fatty acid, dicarboxylate; L-4 = Fatty acid, dihydroxy; L-5 = Inositol metabolism; L-6 = Glycerophospholipid; L-7 = Monoacylglycerol; L-8 = Primary bile acid metabolism; L-9 = Secondary bile acid metabolism; L-10 = Sphingolipid metabolism; L-11 = Sterol/steroid.

By contrast, T3 prostate cancers had increased sphingolipids stearoyl-, euricoyl- and myristoyl- sphingomyelin (*p* = 0.01, 0.02, and 0.04, respectively), as compared with controls. They also displayed lower glycerophospholipid signals for oleoyl-linoleoyl-glycerophosphoinositol (GPI) (OR = 0.49, *p* = 0.000017, which was below the Bonferroni threshold of 0.000027), palmitoyl-linoleoyl-GPI (*p* = 0.001), stearoyl-linoleoyl-GPE (*p* = 0.02), stearoyl-arachidonoyl-GPE (*p* = 0.02), 1-linoleoyl-GPI (*p* = 0.02), and 1-stearoylglycerophosphoglycerol (*p* = 0.03), as well as primary bile acid signals cholate (*p* = 0.002) and chenodeoxycholate (*p* = 0.03), and inositol metabolite inositol 1-phosphate (I1P) (*p* = 0.003) (Table 3).

Also compared to the control group, men with T4 primary tumors showed elevated signals for secondary bile acid lipids taurodeoxycholate (*p* = 0.007), glycodeoxycholate (*p* = 0.007), deoxycholate (*p* = 0.02), tauroursodeoxycholate (*p* = 0.02) and glycolithocholate sulfate (*p* = 0.04), and four sex steroid metabolites [estrone 3-sulfate, 5alpha-pregnan-3beta,20alpha-diol disulfate, 7alpha-hydroxy-3-oxo-4-cholestenoate (7-Hoca), and 4-androsten-3alpha,17alpha-diol monosulfate] (Table 3), as well as several caffeine-related xanthine metabolites (caffeine, 1,3,7-trimethylurate, paraxanthine, theobromine and theophylline) (Table 2). They also had decreased signals for two Krebs cycle metabolites, fumarate and citrate (Table 2).

Of note, we found that a histidine metabolite 4-imidazoleacetate presented an accumulated serum levels from T2 to T4 prostate cancers (OR = 1.28, 1.59, and 2.52, respectively; *p* = 0.11, 0.01, and 0.01, respectively), as well as one secondary bile acid glycolithocholate sulfate (OR = 1.37, 1.35, and 1.83, respectively; *p* = 0.03, 0.07, and 0.04, respectively) (Tables 2 and 3). We found consistent lower levels of glycerophospholipids stearoyl-arachidonoyl-GPE and stearoyl-linoleoyl-GPE in T2 and T3 cases, and a positive signal for euricoyl sphingomyelin in T2 and T3 cases, however, the associations were not seen in T4 cancers (Tables 2 and 3).

We further performed analyses that divided cases by median time to diagnosis for the top signals in T2, T3 and T4 prostate cancers (median time of diagnosis: 8 years, 11 years and 8 years, respectively), and the findings were essentially similar (Supplemental Figures 1–3). We also tested whether the metabolite levels significantly differed across extent of disease category (i.e., heterogeneity) through case-only analyses. We found that metabolites N-Acetyl-3-methylhistidine, 5alpha-androstan-3alpha,17alpha-diol disulfate, deoxycholate and tauroursodeoxycholate showed the lowest *p*_heterogeneity_ (0.00009 ≤ *p*_heterogeneity_ ≤ 0.0029) (Tables 2 and 3).

The findings for T2 and T3 disease do not materially change after adjusting for each of the following factors: trial intervention group, family history of prostate cancer, benign prostatic hyperplasia (BPH), body mass index (BMI), physical activity, cigarettes per day, and serum total PSA, total and high-density lipoprotein (HDL) cholesterol, retinol, and α-tocopherol (data not shown). Because of the small number of T4 cases, the adjusted OR estimates were highly unstable.

## DISCUSSION

The present analysis examining prostate cancer cases with T2, T3 and T4 tumors at diagnosis shows qualitative differences in serum metabolomic profiles up to 20 years prior to clinical diagnosis. The patterns reflective of T2 disease indicate alterations in amino acids, with greater changes in circulating lipid metabolites in men diagnosed with T3 and T4 disease, compared to controls. We found the strongest signals that achieved statistical significance after correction for multiple comparisons were N-acetyl-3-methylhistidine in T2 disease, and the glycerophospholipid oleoyl-linoleoyl-GPI in relation to T3 disease. Thirty metabolites were associated with T4 diagnoses at *p*<0.05, primarily represented by secondary bile acid lipid, sterol/steroid lipid, caffeine-related xanthine metabolites, and Krebs cycle metabolites.

Tumors confined to one or both lobes of the prostate (i.e., T2) were related to serum amino acids in the histidine pathway and those exhibiting N-acetylation. N-acetyl-3-methylhistidine is a post-translational, acetylated derivative of 3-methylhistidine, an important component amino acid residue for both the actin and myosin polypeptides [6]. Histidine and methylhistidine metabolites have been related to tissue remodeling and repair, myofibrillar protein degradation, and changes in the inflammatory response and oxidative stress [7–11]. Increased amino acid acetylation can result from disrupted acetylation activity from aminoacetylase dysregulation, both of which could impact histone-chromatin interactions and gene regulation [12–14]. How such functions relate to the biological/biochemical microenvironment of T2 tumors is unknown, but may indicate active anabolic protein synthesis.

2′-Deoxyuridine was also elevated in T2 cases and has been characterized as a marker for DNA oxidative damage [15], with experimental data showing higher expression in rats exposed to ionizing radiation [16], 5-fluorouracil [17], and high-fat diets [18]. Increased 2′-deoxyuridine could also indicate increased DNA turnover and loss of pyrimidine homeostasis in small, early adenocarcinomas. What role benzoate metabolites might play in these malignancies is unclear.

The serum metabolomic profile of men with tumors extending through the prostatic capsule (i.e., T3) was characterized by reduced signal for several glycerophospholipids, primary bile acid lipids and greater signal for sphingomyelins. The inositol, choline and ethanolamine glycerophospholipids are key cell membrane structural and signaling compounds that provide biosynthetic support during rapid cell proliferation [19, 20]. Sphingomyelins are a primary component in membrane vesicles shed from actively growing prostate and other malignant cells that may facilitate tumor cell invasion and escape from immune surveillance [21–24], and they have been found to be significantly higher in cancer patients than in non-cases [22]. Our finding of elevated serum stearoyl-, euricoyl- and myristoyl- sphingomyelin in T3 cases compared to non-cancer controls (and a smaller elevated risk for the euricoyl-sphingomyelin in T2 cases) is consistent with these prior data. Some of our other findings are of interest as well. Lower serum chenodeoxycholic acid (CDCA), a primary bile acid metabolite, in T3 cases is consistent with lines of evidence indicating that bile acid metabolites including CDCA and its derivatives inhibit malignant cell growth and proliferation through apoptosis induction [25–27], including in prostate cancer cell lines [28–30]. One primary bile acid, cholic acid, also had lower serum concentration in T3 cases.

In the more advanced, T4 cases, lipids remained the most prominent metabolite signals versus controls, but with important qualitative subclass differences compared to men with T3 diagnoses. We found several serum secondary bile acid metabolites were positively associated with T4 cases, including taurodeoxycholate, glycodeoxycholate, deoxycholate, tauroursodeoxycholate and glycolithocholate sulfate (a slightly increased risk for glycolithocholate sulfate in T2 or T3), although none of these associations pass the stringent Bonferroni correction threshold. Earlier studies indicated that bile acids may have a role in carcinogenesis, especially for colorectal cancer, through various mechanisms including tumor promotion and oxidative stress [31, 32]. Deoxycholic acid is metabolized from cholic acid by bacteria in the intestine, and previous study indicated deoxycholic acid can facilitate the growth and progression in colon cancer [33]. Consistent with our findings, an earlier study with 18 prostate cancer cases and 18 controls demonstrated that deoxycholic acid serum levels are significantly elevated in the prostate cancer group when compare to the healthy controls [34]. The underlying mechanisms for bile acids in prostate carcinogenesis will require additional studies. Four sex steroid metabolites were also positively associated with T4 prostate cancer, including estrone 3-sulfate (E3S), 5alpha-pregnan-3beta,20alpha-diol disulfate, 7alpha-hydroxy-3-oxo-4-cholestenoate (7-HOCA) and Δ^4^- and 3α-adiol metabolites. Androgenic activity of, for example Δ^4^-adiol, could be important to maintain growth and possibly androgen independence of the larger and more extensive, T4 tumors [35–37]. Although earlier meta-analysis indicated that coffee consumption may reduce the risk of prostate cancer [38], our metabolomic analysis found higher serum levels of several caffeine-related xanthine metabolites (caffeine, 1,3,7-trimethylurate, paraxanthine, theobromine and theophylline) in T4 prostate cancers, when compared to controls. It is still unclear whether the increased levels of these xanthine metabolites are due to direct biologic roles in T4 tumorigenesis, or result from reverse causality, e.g. increased coffee intake in response to cancer-associated fatigue. This will require further study. We found lower risk of T4 prostate cancer in men with higher serum citrate and fumarate concentration, both of them are intermediates in the Krebs cycle involved with energy production. Earlier studies have demonstrated a higher citrate concentration in the normal prostate peripheral zone glandular epithelium because high zinc ion concentration can prevent conversion of citrate to isocitrate by mitochondrial aconitase [39–41]. The higher citrate concentration may maintain a certain micro-environment in the prostate to provide energy for sperm motility or oocyte fertilization. On the other hand, the signature of malignant prostate cells can be characterized by a low level of zinc and a low level of citrate, which might be possibly reflecting the oxidation of citrate that can yield additional ATP to meet a higher tumor energy requirement, and therefore promote tumor cell anabolism and proliferation [40].

In contrast, we observed that the histamine metabolite 4-imidazoleacetate was progressively positively associated with T3 and T4 prostate cancers (a smaller elevated risk in T2 cases). Histamine metabolites may play a role in mast cell mediated inflammation in prostate carcinogenesis [42]. The metabolite 4-imidazoleacetate usually can be detected in urine samples, and accumulation in serum samples across different T-stages might suggest potential histamine metabolites-prostate cancer associations, which warrants additional examination. Branched chain amino acid (BCAA) metabolism plays an important role in energy production and protein synthesis [43]. Alterations in BCAAs were found to be associated with pancreatic cancer [44], insulin resistance, type 2 diabetes, metabolic disorder, as well as habitual physical activity [45–48]. Of note, leisure-time physical activity has been reported to be positively associated with prostate cancer risk [49], whereas type 2 diabetes was indicated to be inversely associated with prostate cancer [50, 51], although the underlying mechanisms were not well-understood. A previous study suggested that the breakdown of tissue protein may lead to elevated plasma BCAA levels as an early consequence of pancreatic cancer, and BCAAs and/or other amino acids derived from tissue breakdown may contribute to disease progression [44]. By contrast, we did find that the gut microbiota-associated BCAA metabolite 2-hydroxyisobutyrate was associated with risk of T4 disease (T2, T3, and T4 ORs = 0.93, 0.86, and 0.44, respectively; *p value* = 0.6, 0.38, and 0.01, respectively; Table 2). However, the role of BCAA metabolites in the prostate carcinogenesis will require further clinical and prospective studies.

Given that the serum samples were collected up to 20 years in advance of clinical diagnosis, the implications of our study to a large degree rest on whether the observed serologic features represent metabolite patterns reflective of the distinct size and extent of the primary tumors that the individual patients might have transitioned through while still clinically undiagnosed. That is, tumor metabolic effects and requirements for cellular proliferation, membrane signaling and nuclear signal transduction, specific anabolic biosynthetic activities, and mitochondrial ATP production may change with growth of the primary prostate malignancy. Even within each of our tumor stage categories, however, there is likely some degree of metabolic diversity based on the natural history of disease, differences in the time from blood collection to diagnosis, and possibly genetic factors. We have interpreted our findings as reflecting changes in the serum metabolome resulting from the presence of biologically active subclinical prostate tumors of varying size and disease extent. Additional research will be necessary to examine the dynamic changes of these associated metabolites at different time-points of disease development, as well as to evaluate the involving biological pathways that may account for the influence of the associated metabolites on risk of being diagnosed with T2, T3, or T4 prostate cancers.

There are several significant strengths of our investigation. Case ascertainment was based on population registries having high accuracy. The assayed serum samples were collected after an overnight fast up to two decades prior to prostate cancer diagnosis. Case and control samples were stored at −70°C with nearly identical storage durations (and therefore unlikely to have biased our findings), and a high-quality, untargeted metabolomic platform was utilized. Limitations include the relatively homogenous population of Finnish smokers of European descent and the relatively small sample size (particularly with respect to the T4 cases) that may have either precluded identification of other metabolite signals at the Bonferroni or 20% FDR level, yielded false positive findings, or limited additional adjustment for potential confounders.

In conclusion, our investigation of men with prostate malignancies of clinical primary stages T2, T3, and T4 finds evidence of striking qualitative differences in the serum metabolite profiles years in advance of diagnosis. Additional prospective clinical studies would provide useful, alternative tests of the present findings.

## MATERIALS AND METHODS

### Study population

The ATBC Study has been described in detail previously [52]. In brief, the trial enrolled 29,133 Caucasian male smokers aged 50–69 years from southwestern Finland during 1985 to 1988. Men were assigned to one of four intervention groups based on a 2 × 2 factorial design: α-tocopherol (dl-α-tocopheryl-acetate, 50 mg/day), β-carotene (20 mg/day), both supplements, or placebo. Supplementation was for 5–8 years (median = 6.1) until withdrawal from the trial or the end of intervention (April 30, 1993). At baseline, blood samples were obtained from all participants at baseline after an overnight fast and processed to serum, aliquoted, and stored at −70°C; height and weight were measured; and, behavioral and lifestyle information was collected, including smoking, alcohol consumption, medical history, and diet and supplement use.

### Size and extent of primary tumor

The present secondary analysis is based on the aforementioned nested case-control study of prostate cancer [5] that included 71 cases diagnosed with T2 primary cancer (tumor confined within the prostate), 51 cases with T3 (tumor extends through the prostate capsule), and 15 cases with T4 (tumor is fixed or invades adjacent structures other than seminal vesicles, such as external sphincter, rectum, bladder, levator muscles and/or pelvic wall) [53]. Cases in each disease category were compared to a common set of 200 non-case controls. Based on the original case-control set, the controls were matched to the cases on age (± 1 year) and date of baseline blood collection (± 30 days), and were alive and cancer-free at the case diagnosis date. Median time from serum collection to diagnosis was 10 years (range 1–20 years).

### Metabolite assessment

Serum metabolites were measured by Metabolon, Inc. with a high resolution accurate mass (HRAM) platform of ultrahigh performance liquid chromatograph/mass spectroscopy (LC-MS) and gas chromatograph/mass spectroscopy (GC-MS) at Metabolon Inc. as previously described [54, 55]. Procedures included extraction of raw data, peak-identification, and blinded quality control duplicate serum samples included in each batch (8%). We identified 637 known compounds, and after excluding unknown compounds and those for which fewer than 5% of participants had measurable values (*n* = 12), 625 metabolites were retained for analysis which were classified as one of eight mutually exclusive chemical classes: amino acids and amino acids derivatives (subsequently refer to as “amino acids”), carbohydrates, cofactors and vitamins, energy metabolites, lipids, nucleotides, peptides or xenobiotics. The median intra- and inter-batch coefficient of variation across all metabolites was 9% (interquartile range = 4–20%) and 17% (interquartile range = 10–28%), respectively.

### Statistical analysis

Baseline characteristics of the controls and three case sets were compared using *t*-tests for continuous, and chi-squared tests for categorical variables, respectively. We also tested the difference of baseline characteristics across the T2, T3 and T4 case categories using ANOVA tests for continuous variables and chi-squared tests for categorical variables, respectively. The associations between metabolites and case status were based on normalized metabolite signals that divided each value by the batch median followed by log-transformation for normalization. Values below the limit of detection were imputed to the minimum of all non-missing values. Logistic regression models were used to estimate odds ratios (ORs) and their 95% confidence intervals (CI) for the association between a one standard deviation difference in log-metabolite signal strength and prostate cancer risk in each tumor size and extent of disease category (i.e., T2, T3 and T4). Only age (continuous) was retained in the final models after testing and excluding several potential confounders based on a > 10% OR change: trial intervention group, family history of prostate cancer, history of BPH, physical activity, BMI, smoking (cigarettes per day), serum total and HDL cholesterol, serum retinol, and serum α-tocopherol. We further performed additional analyses to subdivide cases (T2, T3 or T4) by the median time to diagnosis, using above-mentioned logistic regression model adjusted for age. We formally tested whether the metabolite levels differed by extent of disease category (i.e., heterogeneity) through case-only analyses. Modeling extent of disease (T2/T3/T4) by polychotomous logistic regression, we report the *p-value* (*p*_heterogeneity_) from a likelihood ratio test comparing models with and without the metabolite level. Based on the Bonferroni correction for 625 metabolites and three tumor classes, the threshold for statistical significance in the main analysis was *p* = 0.000027 [0.05/(625*3)].

Analyses were conducted using SAS software version 9.4 (SAS Institute, Cary, NC, USA). All statistical tests and reported *p* values were two-sided. The trial was approved by institutional review boards of the Finnish National Public Health Institute and the US National Cancer Institute and written informed consent was obtained from all participants.

The other authors declare no conflicts of interest.

## SUPPLEMENTARY MATERIALS FIGURES


