# Supplementary material for: Prospective serum metabolomic profile of prostate cancer by size and extent of primary tumor
Source: Oncotarget. 2017 Apr 1;8(28):45190–9. doi: 10.18632/oncotarget.16775 (PMC5542177; doi:10.18632/oncotarget.16775)
Supplement: Supplementary file 1 [file oncotarget-08-45190-s001.pdf]

Prospective serum metabolomic profile of prostate cancer by size and extent of primary tumor

Supplementary Materials

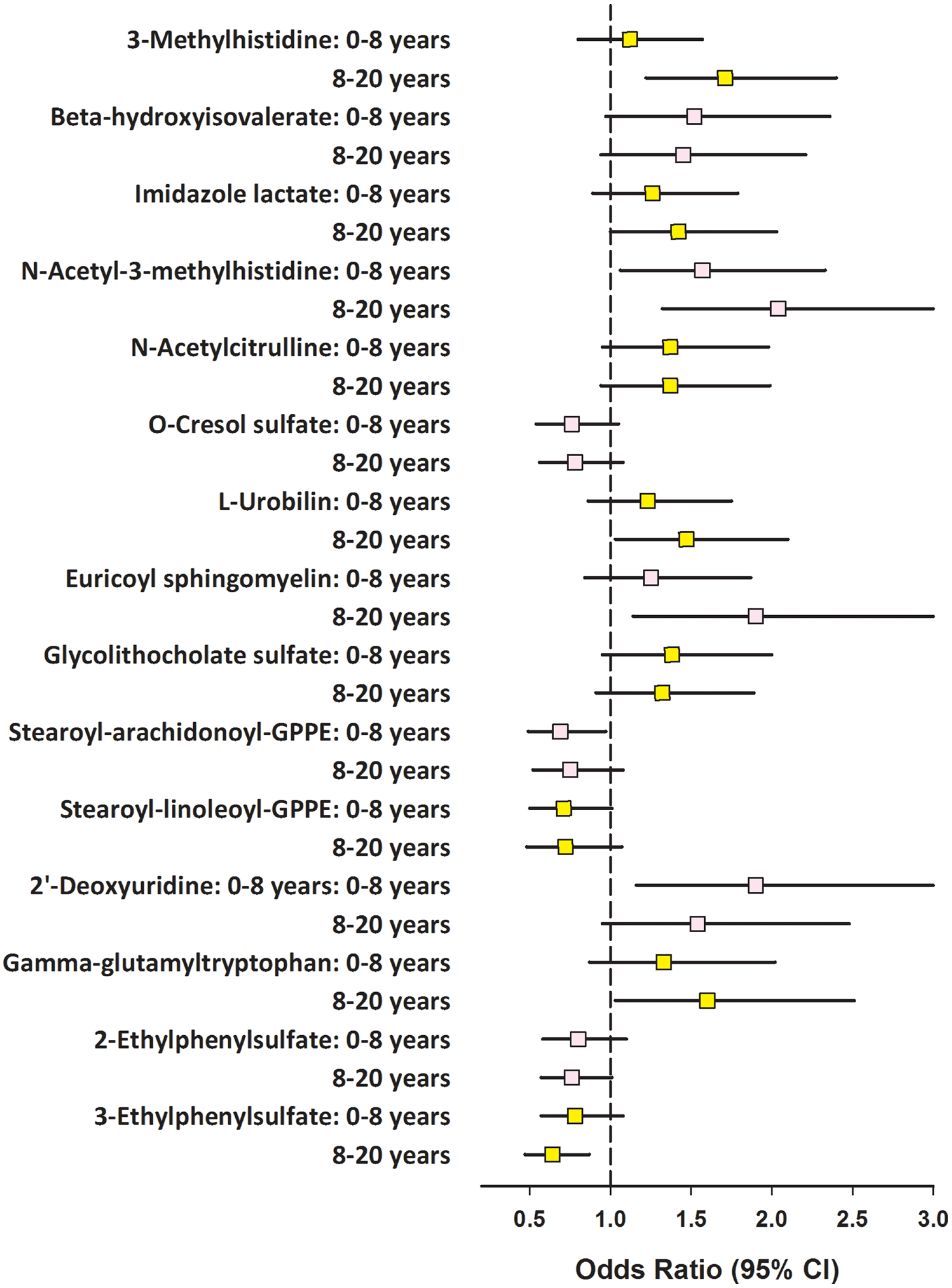

Supplementary Figure 1: Top serum metabolites associated with T2 prostate cancers by median follow-up time.

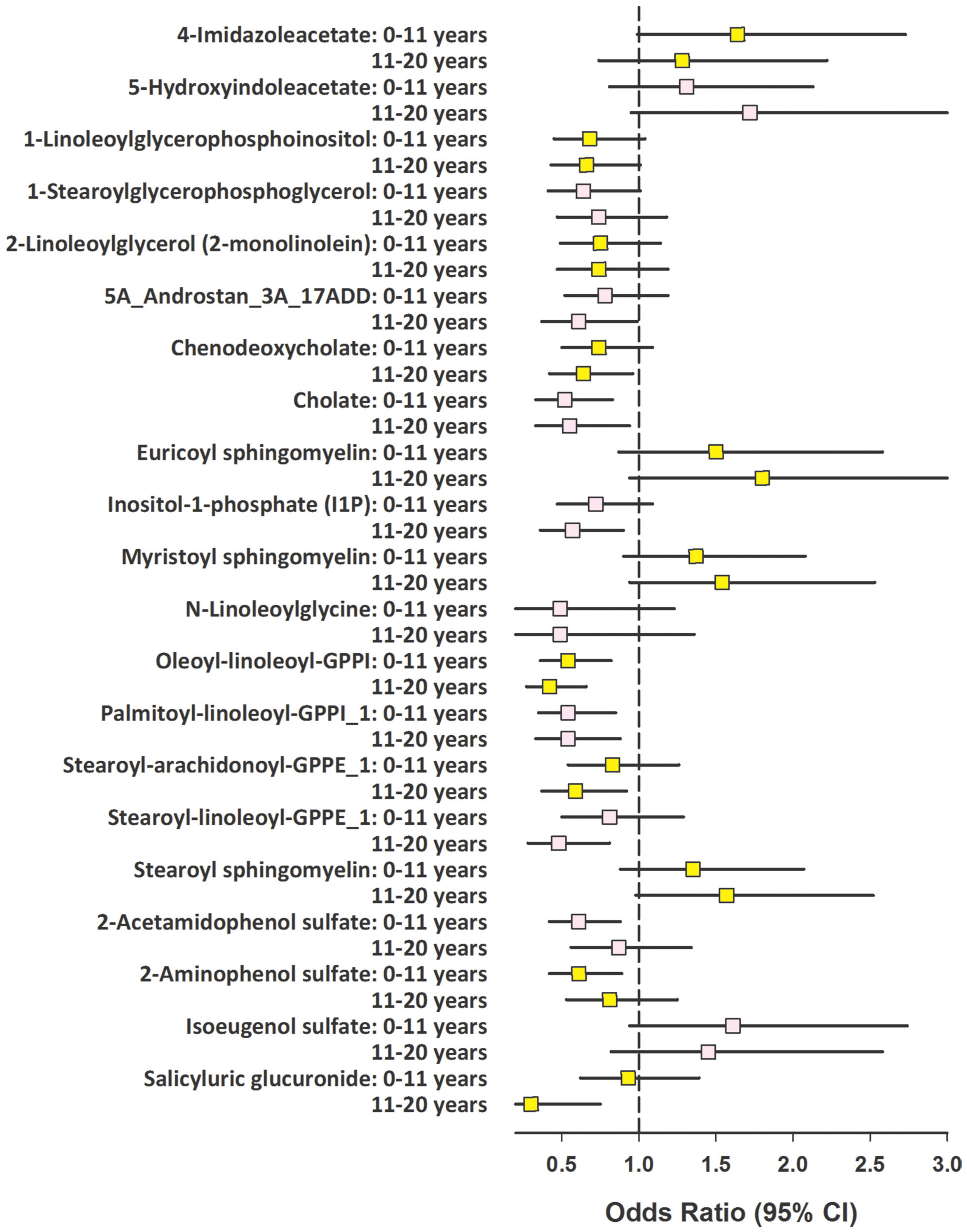

Supplementary Figure 2: Top serum metabolites associated with T3 prostate cancers by median follow-up time.

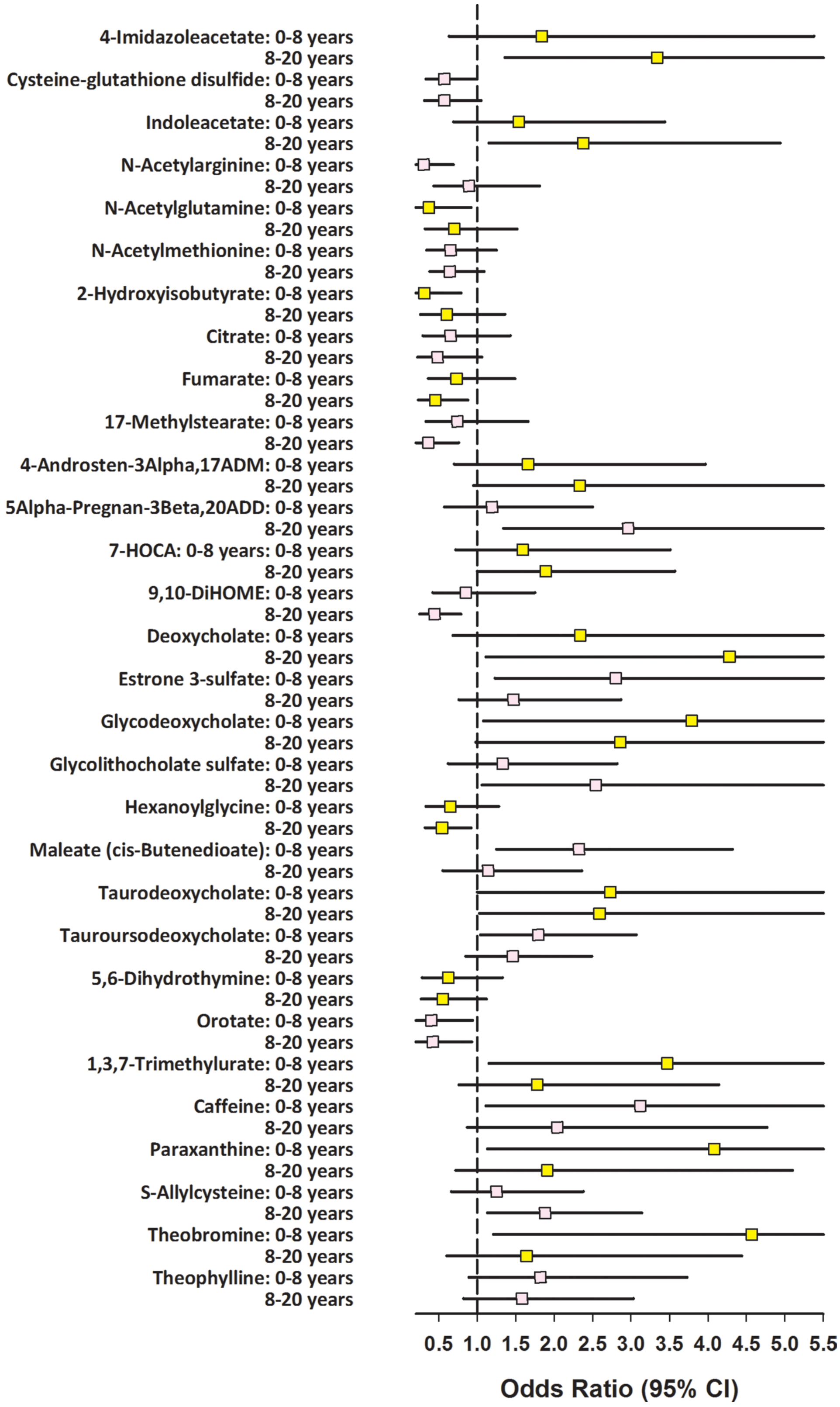

Supplementary Figure 3: Top serum metabolites associated with T4 prostate cancers by median follow-up time.
